# Habitat suitability modelling to assess the introductions of *Aedes albopictus* (Diptera: Culicidae) in the Netherlands

**DOI:** 10.1186/s13071-020-04077-3

**Published:** 2020-04-26

**Authors:** Adolfo Ibáñez-Justicia, Juan Diego Alcaraz-Hernández, Ron van Lammeren, Constantianus J. M. Koenraadt, Aldo Bergsma, Luca Delucchi, Annapaola Rizzoli, Willem Takken

**Affiliations:** 1 Centre for Monitoring of Vectors (CMV), Netherlands Food and Consumer Product Safety Authority (NVWA), Wageningen, The Netherlands; 2 GRECO, Institute of Aquatic Ecology, University of Girona, Girona, Spain; 3 Laboratory of Geo-information Science and Remote Sensing, Wageningen University & Research, Wageningen, The Netherlands; 4 Laboratory of Entomology, Wageningen University & Research, Wageningen, The Netherlands; 5 Research and Innovation Centre, Fondazione Edmund Mach, San Michele all’Adige, Italy

**Keywords:** Invasive mosquitoes, Habitat suitability models, Land surface temperature, MaxEnt, GIS

## Abstract

**Background:**

In the Netherlands, *Aedes albopictus* has been found each year since 2010 during routine exotic mosquito species surveillance at companies that import used tires. We developed habitat suitability models to investigate the potential risk of establishment and spread of this invasive species at these locations.

**Methods:**

We used two methodologies: first, a species distribution model based on the maximum entropy modelling approach (MaxEnt) taking into consideration updated occurrence data of the species in Europe, and secondly, a spatial logic conditional model based on the temperature requirements of the species and using land surface temperature data (LST model).

**Results:**

Suitability assessment obtained with the MaxEnt model at European level accurately reflect the current distribution of the species and these results also depict moderately low values in parts of the Netherlands, Belgium, Denmark, the British islands and southern parts of Scandinavia. Winter temperature was the variable that contributed most to the performance of the model (47.3%). The results of the LST model showed that: (i) coastal areas are suitable for overwintering of eggs; (ii) large areas in the northern part of the country have a low suitability for adult survival; and (iii) the entire country is suitable for successful completion of the life-cycle if the species is introduced after the winter months. Results of the LST model revealed that temperatures in 2012 and 2014 did not limit the overwintering of eggs or survival of adults at the locations where the species was found. By contrast, for the years 2010, 2011 and 2013, overwintering of eggs at these locations is considered unlikely.

**Conclusions:**

Results using two modelling methodologies show differences in predicted habitat suitability values. Based on the results of both models, the climatic conditions could hamper the successful overwintering of eggs of *Ae. albopictus* and their survival as adults in many areas of the country. However, during warm years with mild winters, many areas of the Netherlands offer climatic conditions suitable for developing populations. Regular updates of the models, using updated occurrence and climatic data, are recommended to study the areas at risk.
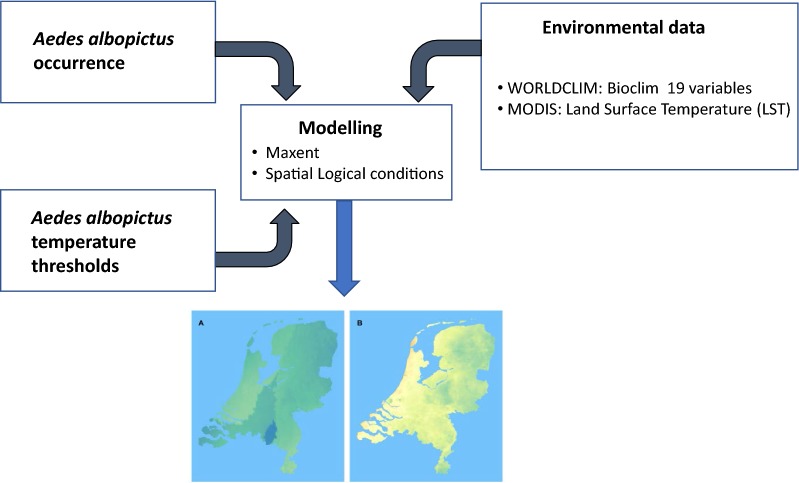

## Background

Increasing international trade and travel facilitate the expansion of the native range of invasive mosquito species (IMS) [1]. The introduction and establishment of IMS are a risk for public health due to the ability of these IMS to transmit vector-borne diseases. Recently, in continental Europe, established populations of the IMS *Aedes albopictus* have been associated with autochthonous cases of several diseases considered “tropical” in Europe, such as chikungunya in Italy [2, 3], dengue and chikungunya fever in France [4–7] and dengue in Croatia, Spain and France [7–9].

*Aedes albopictus* (Skuse, 1895) (Diptera: Culicidae) has its origin in Southeast Asia, but due to global trade and transport the species has expanded its distribution range to all continents of the world except Antarctica [10]. *Aedes albopictus* was introduced into the USA in 1985, most likely *via* import of used tires from Japan [11]. The first sighting in Europe came from Albania in 1979 and probably originated from goods imported from China [12]. However, it was only when the species was introduced into Italy in 1990 through the import of used airplane tires from Atlanta (USA) [13], that the species became established throughout Italy. From the initial introduction location in Italy, the species gradually spread to many European countries [14]. In its native range, *Ae. albopictus* was considered as a rural mosquito [15], breeding preferably in natural habitats and being mainly present at forest edges. Outside its native range, the species has successfully adapted to local conditions and, in many of these areas, became established. The species is characterized by its ecological plasticity and has adapted to breed in various types of man-made water-filled containers (tires, bins, rain barrels, etc.) in suburban and urban areas [16]. *Aedes albopictus* is an undesirable invasive mosquito species (IMS), that causes considerable nuisance by its biting behaviour [17], and the species has been proven to transmit more than 22 different viruses under laboratory conditions [18]. In the field, it is considered a competent vector of chikungunya and dengue viruses [19]. Furthermore, several other pathogens were isolated from specimens collected in the field such as West Nile virus, Eastern equine encephalitis virus [20] and La Crosse virus [21] in North America, and the heartworms *Dirofilaria immitis* and *D. repens* and Usutu virus in Italy [22, 23].

The rapid spread of *Ae. albopictus* across many continents has caused interest in its potential distribution area. Habitat suitability models can be used to investigate the potential risk of establishment and potential spread of this invasive species. Considering its public health threat and invasiveness, several studies have already been conducted at continental scale using habitat suitability models driven by environmental variables [10, 24–29]. Interestingly, the results of these studies at European scale show different outcomes, especially for the northern distribution limits of the species (e.g. UK, the Netherlands, northern Germany). Given these differences among the published predictions thus far, the probability of becoming established following accidental introduction remains unclear for the Netherlands. For example, the studies published for *Ae. albopictus* [10, 24, 27] show a low suitability for the species in the Netherlands compared to southern Europe and the Mediterranean basin. Other studies based on ecological niche models show a moderate suitability in the southern parts of the Netherlands [28, 29]. In the habitat suitability study of Proestos et al. [26], the predicted suitability is high for the Netherlands. A study specifically focused on the Netherlands [30], assessed the possibility for establishment following the accidental introductions in 2005 and 2006 at Lucky bamboo greenhouses [31]. Results of this study suggested that the winter conditions in the Netherlands could be permissive for the establishment of temperate strains of *Ae. albopictus*. In the Netherlands, since 2010, *Ae. albopictus* has been found each year during routine exotic mosquito species surveillance at companies that import used tires [32]. In several locations, the findings occurred at companies that were found positive the year before, suggesting that new sets of used tires containing eggs of *Ae. albopictus* were introduced and stored outside, leading to new findings the following year. Therefore, we investigated whether the species could successfully overwinter at these sites, or whether the species was reintroduced each year again through import of tires containing mosquito eggs. Knowing in advance the locations where *Ae. albopictus* eggs can survive and build up populations after the winter months, helps to decide on a control strategy against the possible populations emerging during the spring.

To address this issue, we created habitat suitability maps for *Ae. albopictus* in the Netherlands using two methodologies. In the first methodology we constructed a habitat suitability map using a species distribution model based on the maximum entropy modelling approach (MaxEnt model), taking into consideration updated occurrence data of the species in Europe (only considering confirmed overwintering of the species at the occurrence location). The MaxEnt approach is one of the most commonly used prediction models to estimate potential spatial ranges of species, and it has recently been used for invasive *Aedes* spp. [29, 33–37]. According to Baldwin [38], the maximum entropy approach is relatively insensitive to spatial errors associated with location data, requires few locations to construct useful results, and performs better than other presence-only models. In the second methodology, we created a habitat suitability map based on a spatial logic conditional model using the temperature requirements of the species measured in other studies, as well as land surface temperature data (LST model). Exploring the habitat suitability of the Netherlands for sustaining populations of *Ae. albopictus* will help to understand the risk for vector-borne diseases associated with the establishment of populations of this species in the country. This may also aid the design of efficient monitoring activities and risk assessments, and lead to more effective methods to prevent establishment of the species given its frequent introductions into the country.

## Methods

### Study area

The spatial extent of the case study area contains the complete territory of the Netherlands: latitude 50.75 N–53.55 N, longitude 3.35 W–7.22 E. The study area is characterized by a flat landscape with large areas occupied by lakes, rivers and canals. The overall study area accounts 34,933 km^2^ not including the inland waters. About 25% of the land is at, or below sea level. The climate is temperate, with mild winters, cool summers, and rainfall in every season. Urban and peri-urban areas represent approximately 20% of the country total area [39].

### MaxEnt model

Presence data for *Ae. albopictus* in Europe was taken from Cunze et al. [29] and included occurrence locations from Kraemer et al. [33] and Koch et al. [35] until 2015. This database was verified according to maps such as the European Centre for Disease Prevention and Control (ECDC) [40], Entente Interdépartementale de Démoustication (EID) Méditerranée [41], Collantes et al. [42], and Prioteasa et al. [43], using the georeferencing tool of ArcGIS software. Mosquito records without confirmed establishment or overwintering were excluded from the dataset. Also 138 new occurrence records were added using these maps. After verification, the occurrence dataset in Europe accounted for 426 records. An additional map shows the occurrence records in more detail (see Additional file 1: Figure S1).

Bioclimatic data were used at a spatial resolution of 2.5 minutes (~ 4.5 km at the equator) for our MaxEnt model. Nineteen bioclimatic variables were downloaded as raster files from WorldClim (http://www.worldclim.com) for the European extent: latitude, 28.98 N–73.22 N; longitude, − 16.30 W–48.25 E. For the selection of these variables we performed a correlation analysis following the next steps. First, we merged around each occurrence record (426 occurrences) a buffer with 200 km of radius. Secondly, we generated 10,000 random background points within this accessible buffer area. Finally, we used the occurrence locations and the values of the 10,000 random background for the correlation analysis. Variables that were not strongly correlated with a Pearson’s correlation coefficient less than 0.8 and were ecologically relevant for the species were chosen for the model (see Additional file 1: Figure S2). In order to avoid potential collinearity problems within the set of variables used in the model, we assessed the collinearity with the variance inflation factor (VIF), which is a measure of correlation between pairs of variables. The VIF was calculated for all combinations of variables and the selected variables presented a collinearity value < 7 (see Additional file 1: Figure S3). Correlation and collinearity analysis were performed in R version 3.5 [44]. We used five bioclimatic variables from the 2.0 WorldClim dataset (time series 1970–2000): BIO2, mean diurnal range (mean of monthly (maximum temperature − minimum temperature)); BIO7, temperature annual range (maximum temperature of warmest month − minimum temperature of coldest month); BIO8, mean temperature of the wettest quarter; BIO11, mean temperature of coldest quarter; and BIO12, annual precipitation.

We calibrated a preliminary set of models using the Kuenm_ceval function implemented in the *Kuenm* R package [45]. The purpose of the calibration is to evaluate the best potential combination of selectable parameters in MaxEnt to select the most appropriate model (feature classes, regularization multiplier and bioclimatic variables). For the calibration we used the presence and background data locations where 75% of the records were used for training the model and 25% for the test. Candidate models were created, with parameters reflecting all combinations of 17 regularization multiplier settings (0.1, 0.2, 0.3, 0.4, 0.5, 0.6, 0.7, 0.8, 0.9, 1, 2, 3, 4, 5, 6, 8 and 10), 29 feature class combinations (logistic, quadratic, product, threshold, hinge) and 26 distinct sets of environmental variables. These models were later evaluated based on statistical significance (Partial ROC), omission rates (OR) and Akaikeʼs information criterion corrected for small sample sizes (AICc). The model with lower AICc and OR value was chosen among all candidate models as the best model. We then used 100 replications to run this best model using the above-mentioned parameters.

The modelling performance was evaluated using the AUC-value. Area under curve (AUC) measures the sensitivity of the model and was used to test the performance of the model with real observations in the training area. An AUC value of 0.5 shows that the model predicts randomly, while a value close to 1 indicates optimal model performance. The relative contributions of the five environmental variables to the MaxEnt model were estimated using permutation importance estimation.

### Land surface temperature model

For the LST model, we used the 4×-daily 250 m LST reconstruction of Metz et al. [46]. The dataset was generated from twice-daily 1 km overpasses from the Terra and Aqua sensors onboard NASA’s Moderate Resolution Imaging Spectroradiometer (MODIS), using a method of weighted temporal averaging, statistical modelling and spatial interpretation [46]. We computed and used daily averages spanning 1/1/2009–31/12/2015 (Table 1). The data was provided by the Edmund Mach Foundation (San Michele all’Adige, Italy).

**Table 1:** Data frame environmental variables used in the MaxEnt and LST model

Features	MaxEnt model	LST model
Source	WorldClim	MODIS sensor
Environmental variables	Bioclimatic variables	Land surface temperatures
Temporal frequency	Monthly	Daily
Temporal extent	1-Jan-1970 until 31-Dec-2000	1-Jan-2009 until 31-Dec-2015
Spatial resolution	2.5 minutes (about 4.5 km at the equator)	250 meters
Spatial extent	Europe Latitude: 28.98 N–73.22 N Longitude: - 16.30 W–48.25 E	The Netherlands Latitude: 50.75 N–53.55 N Longitude: 3.35 W–7.22 E

For this model we applied the workflow followed by Neteler et al. [47], to turn the processed daily MODIS LST data into ecological indicators that can be used to assess the potential spatial distribution of *Ae. albopictus*. From this dataset, we derived the following predictors for the period between 2009–2015 in the Netherlands: mean temperature of January, the mean annual temperatures and accumulated daily growing degree days (GDD). These were first calculated per year. The overall probability averages were calculated based on the yearly probabilities between 2009–2015. The output indicators (i) probability of overwintering of eggs (POE), (ii) probability of adult survival (PAS), and (iii) probability of life-cycle completion (PLC) were predicted. We adopted the methodology and the threshold values used by Neteler et al. [47], based on the studies of Kobayashi et al. [47], Roiz et al. [49] and Caminade et al. [25] as follows: (i) the threshold for the POE was set to 1 °C for the mean January temperature with a margin of 2 °C (between − 1 and 3 °C); (ii) the threshold for the PAS was set to 11 °C for the mean annual temperature with a margin of 2 °C (9–13 °C); and (iii) the threshold for PLC based on 1350 GDDs and 11 °C, was set to 1st September, with a margin of one month. The GDDs are defined as the degrees exceeding a given threshold (11 °C for *Ae. albopictus*) accumulated for all days in a given year [47]. The model workflow used a gradient of suitability that considers uncertainty in the LST data [50] as well as spatial uncertainty. The calculated gradients range from 0 (unsuitable) to 1 (highly suitable). As in the study of Neteler et al. [47], areas with suitability values between 0 and 1 are moderately suitable and *Ae. albopictus* may or may not survive in these areas. Spatial data processing was performed with ArcGIS Pro.

### Interpretation of the model results using IMS surveillance data

The suitability maps obtained with MaxEnt and LST models were then contrasted with the findings of *Ae. albopictus* during surveillance in used tire companies (2010–2015) to establish relationships considering the possibility of overwintering and survival of the species at these locations. Furthermore, we included an additional analysis of the LST data registered during the winter at the used tire locations based on the results of the laboratory experiments of Thomas et al. [51]. These results showed that eggs of European *Ae. albopictus* could resist temperatures of − 10 °C for at least 24 h and even show low rates of hatching after exposure to temperatures as low as − 12 °C for short periods. Used tire locations and years showing daily temperatures below − 10 °C were extracted and interpreted. As daily LST data are not directly comparable to the air temperature thresholds measured in the laboratory, we adjusted the winter LST data to air temperature at these used tire locations. First, we downloaded daily average temperature values of 20 weather stations across the Netherlands from 2010 until 2015. Secondly, we investigated the linear correlation between winter LST values (December, January and February) and air temperature values from the weather stations. If the correlation coefficient was > 0.8, we obtained the equation to adjust the winter LST data to air temperature using a regression model. Finally, we adjusted the winter LST data to air temperature at the used tire locations to detect possible exposures of *Ae. albopictus* eggs to lethal temperatures. The habitat suitability values of both models (value between 0–1), and the LST data were extracted for 11 occurrence locations at the used tire companies by a raster to point operation (QGIS v.2.18.14). Georeferenced data from surveillance of invasive mosquito species collected by the Centre for Monitoring of Vectors of the Netherlands Food and Consumer Product Safety Authority was used for this assessment (see Additional file 1: Figure S4). Probability values obtained at the finding locations with the MaxEnt and with the LST model (probability of overwintering of eggs (POE), probability of adult survival (PAS) and probability of life-cycle completion (PLC)) were interpreted comparing the results between the finding locations.

## Results

In our study, we predicted the potential areas suitable for *Ae. albopictus* populations in the Netherlands using two modelling approaches. The MaxEnt model predicted the habitat suitability using current occurrence data of the species in Europe and bioclimatic variables derived from monthly temperature and precipitation values (Table 1). In the LST model, the habitat suitability was estimated based on temperature thresholds for *Ae. albopictus* survival and establishment. We found differences in the predicted suitability values between the two models.

**Fig. 1:**
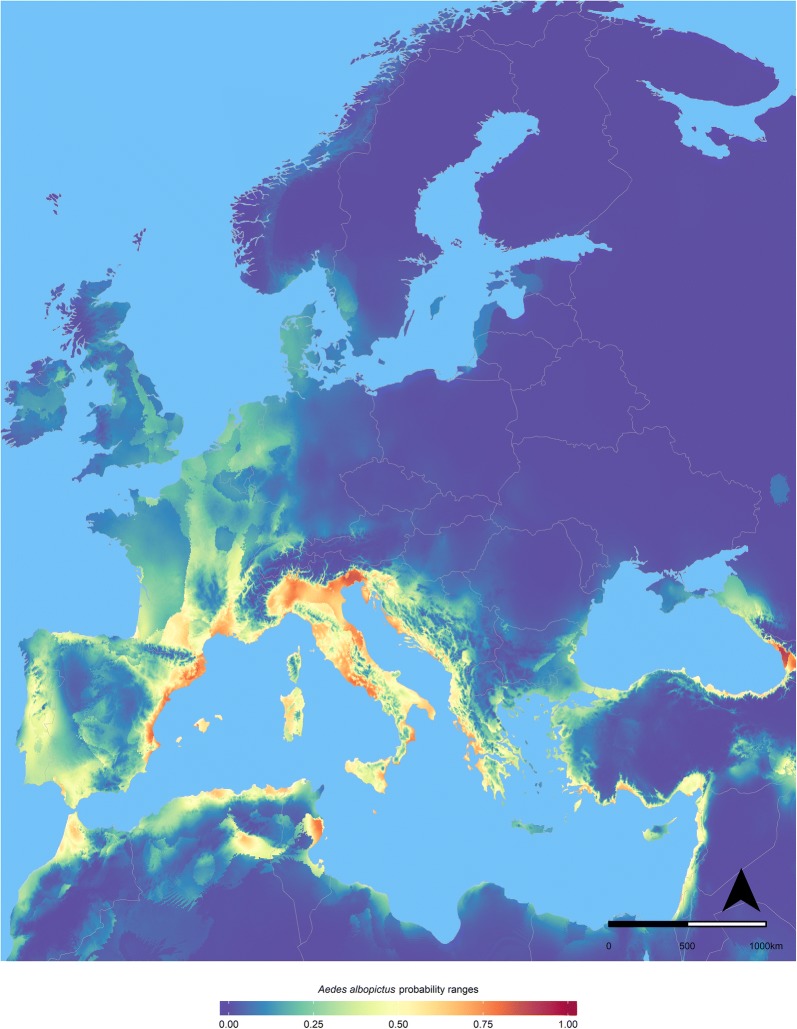
Modelled European habitat suitability for *Ae. albopictus* with MaxEnt expressed by probability range. Map by authors

**Fig. 2:**
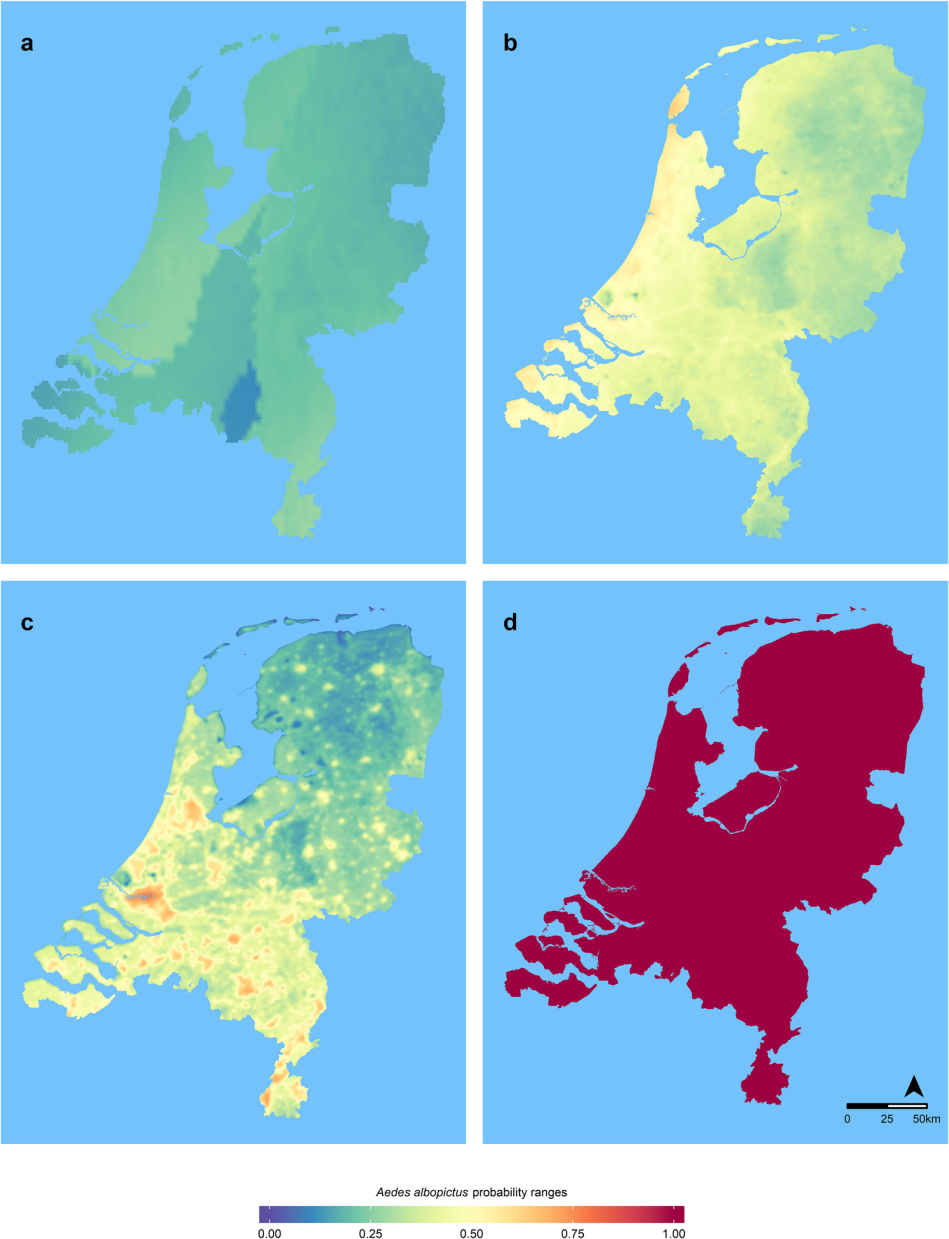
Modelled habitat suitability for *Ae. albopictus* in the Netherlands expressed by probability ranges obtained with the MaxEnt model (**a**) and the Spatial Logical Condition model for land surface temperature (LST) (years 2009–2015) and considering overwintering eggs (POE) (**b**), adult survival (PAS) (**c**) and life-cycle completion (PLC) (**d**)

Results obtained with the MaxEnt model at European level accurately reflect the observed current distribution of the species. The model accounted an AUC value of 0.81 ± 0.009. Modelling results show high probability of suitable conditions for *Ae. albopictus* in southern Europe, especially in the Mediterranean parts of Spain, France and Italy, and along the Adriatic shore (Fig. 1). Our model depicts high suitability also in the western part of the Iberian Peninsula, the Rhone and parts of the Rhine valleys in France, Switzerland and Germany. The model also depicts moderately low values in parts of the Netherlands, Belgium, Denmark, the British islands and the southern parts of Scandinavia. In the Netherlands, the relative climatic suitability can be considered moderately low as expressed by the values ranging between 0.11 in the south of the country and 0.28 in the coastline (Fig. 2a) and in comparison to the suitability of the Mediterranean coast with values higher than 0.80 (Fig. 1). Underlying statistics like maximum, median and standard deviation obtained with the MaxEnt model are available (see Additional file 1: Figure S5). According to the estimated permutation importance, our results show that winter temperature (temperature of the coldest quarter of a year) was the variable that contributed most to the model performance (47.3%), while the mean temperature of the wettest quarter and the annual precipitation contributed with 23.6% and 13.3%, respectively (Table 2). The relative contribution of five environmental variables to the model according to the regularized training gain of the jackknife test is presented in Additional file 1: Figure S6. Results of the response curves showing the relationships between the probability of the presence of *Ae. albopictus* and five environment variables are presented in Additional file 1: Figure S7.

**Table 2:** Average percentage (%) of the relative contributions and permutation importance of the most important environmental variables into the *Aedes albopictus* MaxEnt model

Code	Variable	Contribution	Permutation importance
BIO11	Mean temperature of coldest quarter	39.67	47.33
BIO8	Mean temperature of wettest quarter	27.55	23.64
BIO12	Annual precipitation	13.67	13.31
BIO7	Temperature annual range	13.85	10.01
BIO2	Mean diurnal range	5.26	5.70

*Notes*: BIO11, mean temperature of coldest quarter; BIO8, mean temperature of the wettest quarter; BIO12, annual precipitation; BIO7, temperature annual range (maximum temperature of warmest month − minimum temperature of coldest month); BIO2, mean diurnal range (mean of monthly (maximum temperature − minimum temperature)

The overwintering ability of the diapausing eggs has been related to the January mean temperature in the Netherlands in the LST model. Considering the averaged January mean temperature from 2009 to 2015, nearly all inland parts of the country show a low predicted habitat suitability for diapausing eggs of *Ae. albopictus* (Fig. 2b). The model predicts moderate suitability for egg overwintering in the areas along the major rivers in the Netherlands (e.g. Maas, Rijn, IJssel and Maas). The areas along the coast show a higher suitability for egg overwintering, especially the island of Texel and surroundings in the utmost north-western part of the country. In the LST model, adult survival is determined by mean annual temperatures. Results obtained with the temperatures show that the whole country is suitable for successful life-cycle completion (Fig. 2d) and that suitable areas for adult survival are less restricted than the overwintering areas (Fig. 2c). However, large areas in the northern part of the country have a low suitability for adult survival. Highly suitable areas for survival of adult mosquitoes are the surroundings of the harbour of Rotterdam, the surroundings of Amsterdam and large areas in the provinces of Limburg and Noord-Brabant, mostly associated with urban centres (e.g. ‘s-Hertogenbosch, Eindhoven, Tilburg, or Maastricht) (Fig. 2c).

The suitability values obtained with MaxEnt and LST models were further interpreted with direct observation data from mosquito surveillance at the used tire companies in the Netherlands. Suitability values calculated for the locations where *Ae. albopictus* has been detected during surveillance are presented in Table 3. Habitat suitability values obtained with the MaxEnt model ranged from 0.26 in Moerdijk to 0.17 in Hardenberg. According to the MaxEnt model results, we assume that the winter temperature in the Netherlands is the variable that constrains the survival of the diapausing eggs, thereby reducing the chance of establishment of the species. Considering the average January temperature from all the years at the used tires locations in the LST model, *Ae. albopictus* does not encounter conditions that would completely limit the overwintering of diapausing eggs at these locations (Table 3). For all locations where the species was found outdoors, the mean probability for overwintering of eggs ranged between 0.3–0.5. Furthermore, when maps are considered per year rather than the 7-year average, January temperatures in 2012 and 2014 did not limit the overwintering of eggs at the locations where the species was found the previous year. Linear correlation was obtained (correlation coefficient = 0.83) using the winter air temperature from weather stations and LST data (see Additional file 1: Figure S8), and we used this correlation to correct the LST data to air temperature in the winter months. Using the corrected daily average LST below - 10 °C as a threshold for *Ae. albopictus* egg-hatching after the winter, we did not find any used tire location with an average temperature of below - 9 °C during the winter months of any sampling year. However, using the suitability gradient of the POE, overwintering of eggs is not considered likely in 2010, 2011 and 2013, and the presence of the species could be attributed to new introductions of specimens (eggs) at the tire importers in the next spring or summer. In 2014, annual temperatures indicated a high probability for survival of adults at the locations where *Ae. albopictus* was found (Table 3). Considering the average temperature from 2009 to 2015, at the location Weert, the species also encounters suitable conditions for adult survival (P = 67%, Table 3). Using the same threshold, *Ae. albopictus* does not clearly encounter conditions for adult survival at several locations. Findings at locations in Emmeloord, Montfoort, Assen, Moerdijk, Lelystad and Almere accounted low probability for adult survival (P ≤ 40%). Furthermore, the annual temperatures in 2010, 2012 and 2013 also limited the conditions for survival of adults in several locations (Table 3).

**Table 3:** Results obtained with the LST models and MaxEnt model for *Aedes albopictus* at the outdoors finding locations in the Netherlands (years 2010–2015)

Location	2010			2011			2012			2013			2014			2015			Mean POE	Mean PAS	Mean MaxEnt
	POE	PAS	PLC	POE	PAS	PLC	POE	PAS	PLC	POE	PAS	PLC	POE	PAS	PLC	POE	PAS	PLC			
1. Almere	0.000	0.000	1.000	0.370	0.415	1.000	0.645	0.202	1.000	**0.000**	**0.184**	**1.000**	1.000	0.654	1.000	0.669	0.449	1.000	0.393	0.308	0.242
2. Assen	0.000	0.000	1.000	0.265	0.445	1.000	0.557	0.276	1.000	**0.000**	**0.267**	**1.000**	**0.962**	**0.706**	**1.000**	**0.501**	**0.549**	**1.000**	0.326	0.376	0.174
3. Emmeloord	0.000	0.000	1.000	0.410	0.485	1.000	0.781	0.325	1.000	**0.062**	**0.302**	**1.000**	**1.000**	**0.761**	**1.000**	**0.690**	**0.564**	**1.000**	0.420	0.403	0.211
4. Etten-Leur	0.000	0.000	1.000	0.492	0.567	1.000	0.748	0.312	1.000	0.000	0.317	1.000	1.000	0.694	1.000	**0.581**	**0.564**	**1.000**	0.403	0.401	0.190
5. Hardenberg	0.000	0.000	1.000	0.267	0.407	1.000	0.513	0.261	1.000	**0.000**	**0.277**	**1.000**	**1.000**	**0.652**	**1.000**	0.410	0.429	1.000	0.312	0.347	0.172
6. Lelystad	0.000	0.000	1.000	0.388	0.395	1.000	0.815	0.254	1.000	**0.117**	**0.218**	**1.000**	**1.000**	**0.694**	**1.000**	**0.720**	**0.457**	**1.000**	0.434	0.326	0.233
7. Moerdijk	**0.000**	**0.000**	**1.000**	0.663	0.511	1.000	**0.888**	**0.308**	**1.000**	0.105	0.238	1.000	1.000	0.640	1.000	0.751	0.471	1.000	0.486	0.361	0.264
8. Montfoort	**0.000**	**0.000**	**1.000**	0.431	0.481	1.000	**0.827**	**0.281**	**1.000**	**0.000**	**0.266**	**1.000**	1.000	0.691	1.000	**0.693**	**0.499**	**1.000**	0.421	0.372	0.259
9. Oosterhout	0.000	0.048	1.000	**0.420**	**0.636**	**1.000**	0.767	0.404	1.000	0.004	0.401	1.000	1.000	0.776	1.000	0.525	0.627	1.000	0.388	0.470	0.188
10. Oss	**0.000**	**0.119**	**1.000**	**0.435**	**0.713**	**1.000**	**0.768**	**0.486**	**1.000**	**0.157**	**0.572**	**1.000**	**1.000**	**0.874**	**1.000**	0.679	0.746	1.000	0.434	0.588	0.209
11. Weert	**0.000**	**0.212**	**1.000**	**0.151**	**0.806**	**1.000**	**0.761**	**0.538**	**1.000**	**0.083**	**0.694**	**1.000**	**1.000**	**0.914**	**1.000**	0.786	0.869	1.000	0.397	0.669	0.225

*Note:* Numbers in bold indicate the probability results in relation to the findings at that specific location and year

*Abbreviations*: POE, probability for overwintering eggs; PAS, probability for adult survival; PLC, probability for life-cycle completion

## Discussion

The findings of the invasive mosquito species *Ae. albopictus* in used tire companies in the Netherlands demonstrate the long-distance human-aided dispersal capabilities of this species [52]. Given its potential as a vector of infectious diseases, the question that arose was whether this non-native mosquito species could survive under the climate conditions of the Netherlands, and whether it would be able to establish permanent populations in this country. In this study, we predicted the potential areas that may be suitable for *Ae. albopictus* populations in the Netherlands using two modelling approaches. Results showed differences in the predicted suitability values between the models. The MaxEnt model was the more restrictive for the species in the Netherlands. The winter temperature is the variable that most contributes to the performance of the model, thereby reducing the chance of establishment of the species in northern parts of Europe. However, we have found the species outdoors at used tire companies every year since 2010 at different locations across the country. These findings can be explained by the continuous and frequent introduction of used tires at the facilities containing *Ae. albopictus* diapausing eggs from areas where the species has established populations (e.g. northern Italy or southern France). Conversely, the LST model showed that coastal areas are suitable for overwintering of eggs and the entire country is suitable for successful completion of the life-cycle if the species is introduced after the winter months.

The MaxEnt results showed lower habitat suitability than the LST model probably due to: (i) the earlier time-frame temperature period used on the MaxEnt model (MaxEnt: 1970–2000; LST: 2009–2015); and (ii) the use of two different datasets, one based on LST data and another that uses air temperature to compute the temperature-based bioclimatic variables. According to the Royal Netherlands Meteorological Institute (KNMI) climate scenarios [53], the area of the Netherlands experienced a change of monthly temperature increase that has favoured the invasive mosquito since 2000. Additionally, in the MaxEnt model, the time period of the climatic data used for training (1970–2000) does not widely match the time period of the used occurrence data. First records of populations of the species in Europe (Italy) date back to the early 1990’s, but for example records in Spain and Germany were reported after the year 2000 [54, 55]. Therefore, we assume that if the species would have been introduced before the year 2000 in these areas, it would have successfully become established due to the proven invasiveness of *Ae. albopictus*. Unlike other similar models [29, 34], our MaxEnt model has only been fed with known current established populations or locations where overwintering of the species has been observed and reported. These studies were performed using also occurrence data from interceptions without confirmation of establishment or overwintering (e.g. interceptions in Lucky bamboo greenhouses in the Netherlands). In our opinion, results of these models could lead to an overestimation of the northern limits of the species in Europe.

As observed in previous species distribution models [25, 29, 37], an expansion of climatically suitable habitats in western and central Europe is expected in the near future for *Ae. albopictus*. In our study, climate change scenarios have not been taken into account. As known from the climate scenarios developed by the KNMI, even under conservative and optimistic scenarios, the temperature in the Netherlands is expected to continue to rise, with an increase of values between 1–2.3 °C in 2050 and between 1.3–3.7 °C in 2085 [53]. That means that climate change will undoubtedly increase the winter temperatures, leading to the increase of the probability for overwintering of eggs, and consequently increasing the risk of establishment of *Ae. albopictus* in some parts of the Netherlands within 30 years.

The LST model showed very low suitability values in forested areas and greenhouse farming houses and the very high values in urban areas (e.g. built-up areas, residential, industrial). These results are in accordance with Guo et al. [56] who found that built-up areas with paved roads, residential and factory buildings have a higher LST, and vegetated land covers have the lowest LST. A possible explanation for a low LST in vegetated areas is that the LST is collected from the forest canopy, which is influenced by the cooling effect of evaporation. The low suitability accounted in the greenhouse buildings situated in the western part of the country, is directly related to the highly reflective roofs of the greenhouses, an artefact which suggests that these locations are cool places.

The results of our study provide a new perspective on the previous study of Takumi et al. [30]. The latter study was based on climatological information from weather stations (only from 2006) and concluded that winter conditions in the Netherlands were permissive for the establishment of temperate strains of *Ae. albopictus*. In our study, two parameters that act against the establishment of *Ae. albopictus* in areas where diapausing eggs may overwinter, were also taken into account: the annual mean temperature (11 °C), and the day of year when 1350 growing degree days (GDDs) are reached. These parameters are considered important because they affect the time needed for *Ae. albopictus* larvae to mature into adult mosquitoes during the period of seasonal activity. It should be mentioned that in comparison with the study of Takumi et al. [30], we did not use precipitation data in the LST model. For optimal development of *Ae. albopictus*, the species requires at least 500 mm of annual rainfall [57]. The mean annual rainfall in the Netherlands ranges from about 700 to 900 mm [58] and is not considered a limiting factor for the species to be taken into account in our LST model.

In our LST model study, the results depend on the selected thresholds for the January temperature (1 °C), annual temperature (11 °C), and day of year when 1350 GDDs are reached. These threshold values were based on the studies of Kobayashi et al. [48], Roiz et al. [49] and Caminade et al. [25]. Modifying these temperature thresholds with a few degrees could result in a substantial change in the suitable area for the species. For example, lowering the January and annual temperature thresholds a few degrees would increase the area regarded as suitable for *Ae. albopictus*. In the same way, moving by several weeks before the threshold value when 1350 GDDs are reached may result in a decrease in the suitable area. We based our LST model study on temperature thresholds studied in other areas of the world. For the temperate strain of *Ae. albopictus* spreading in Europe, the threshold of 11 °C has been accepted as lower threshold for larval growth in the USA [59] and in Japan [48]. Furthermore, in the risk maps produced by the European Centre for Disease Prevention and Control (ECDC) in 2009 [24], it was concluded that the criterion of an annual mean temperature of 11 °C seemed to fit well with the overall observed distribution of the temperate strain of *Ae. albopictus* in Europe. Compared with results from studies in Italy [60] and Switzerland [47], our GDDs model results do not reveal new suitability hotspots for the species in the Netherlands. As shown in the results, the species could develop from larvae to adult every year in the whole study area for the period 2009–2015.

As stressed by Thomas et al. [51] and Tippelt et al. [61], single events of extreme temperatures in short periods may have a strong impact on overwintering of *Ae. albopictus* because they can cause irreversible damage to the eggs. Laboratory experiments of Thomas et al. [51] have shown that eggs of European *Ae. albopictus* could endure temperatures of - 10 °C for at least 24 h and even show low rates of hatching after exposure to temperatures as low as - 12 °C for short periods. During the study period, our results show that none of the used tire locations was affected by short periods of temperatures below - 10 °C. This suggests that at these locations, *Ae. albopictus* eggs were not exposed to single events of critical temperatures that could affect hatching rates after winter, and in turn could affect the production of sufficient individuals to build up a population during the next season.

In the Netherlands, after the implementation of a risk-based surveillance strategy on invasive mosquitoes, *Ae. albopictus* has been intercepted yearly at used tire companies or its surroundings [32]. However, the species has not successfully established in these interception areas. Because surveillance and control of invasive mosquitoes started in 2010, and used tire companies had already imported tires before the start of the surveillance, *Ae. albopictus* may have been introduced many years before 2010. The question is whether in the absence of surveillance and control measures, what factors could have worked against the successful establishment in these areas. The MaxEnt results indicate low suitability for the species in the Netherlands, in comparison with areas in southern Europe, and only one location (Moerdijk) in the LST model results showed moderate suitability for overwintering of eggs (value < 0.5). The length of the reproductive season in the Netherlands could also contribute to lowering the probability of suitable conditions. In fact, this length is considerably shorter in the Netherlands than at southern latitudes in Europe, due mainly to differences in temperature. This means that the temperature necessary to stimulate the hatching of diapausing eggs will occur later in the season in the Netherlands than in the southern European regions, and the temperature causing adult mortality will arrive early in the season. However, *Ae. albopictus* is a species with a high ecological plasticity that can cope with a wide range of climatic conditions, has competitive ability, and has demonstrated to have the potential to adapt fast to the environment during the invasion process [62]. All these facts might allow *Ae. albopictus* to adapt to colder temperatures in the new invaded locations, facilitating establishment in colder regions. Considering the applied methodological approach and the used data, the physiological plasticity of the species [62] can be considered as the most limiting factor affecting the prediction in both models. In MaxEnt, if in the future the species will become established in drier environments (i.e. in southern Spain), or in colder northern regions (i.e. Belgium or the Netherlands), the contribution of these occurrence locations used in MaxEnt will result in a different output than our study. In the LST model, the adaptation to colder temperatures will make the use of survival temperature thresholds not useful. Possible adaptation to colder climates will increase the areas suitable in northern regions.

We conducted our study using climatic variables exclusively as the main factors effecting the distribution of *Ae. albopictus* in the Netherlands. However, other factors such as land cover/land use [63], habitat availability and microclimate [64] can significantly influence the success of the establishment in an area. In the case of the Netherlands, the winter and annual temperatures are the limiting factors in our models. In this case, hibernation of diapausing eggs indoors or in warm and protected areas in cities, may protect the mosquito from cold events and may be responsible for local establishments under these special conditions. Especially the effect on the microclimate created by the tires could not be assessed within the framework of this study but is potentially influential. We believe that the tires can provide a suitable site for egg overwintering, larval development and adult shelter. Several factors can influence the microclimate in the tire locations, such as storage method (piles, pyramids, etc.), amount and type of tires, or exposure to wind. As also shown in our results, higher temperatures can be expected around cities (heat island effect) [63]. In cities, potential breeding sites such as urban catch basins can provide favourable microclimatic conditions for overwintering of diapausing eggs compared to more cold-exposed sites [64]. Unfortunately, these are factors that could not be considered in this study and are recommended for future investigations. Because the species is currently invading areas northwards in Europe [40], regular updates of the modelling using updated occurrence and climate data are recommended.

In summary, the present results lead to a better understanding of the species’ potential distribution and identified areas with a risk for the establishment of the species in the Netherlands. However, the results of the two modelling approaches were different, and for the interpretation of these results, one needs to be aware of the limitations of both modelling approaches. For the MaxEnt model, regular updates of the model using the most recent occurrence and climate data are recommended. Specifically, new occurrence data on established populations in northern regions in Europe coupled with climate data corresponding to the occurrence period (1990–2018) will be of interest to accurately predict the geographical limits of the species. For the LST model, yearly updates of the model after the winter months, and using the recent data will provide the egg overwintering probability in the study area, including also the used tire locations where the species was found before the winter months. This information will be relevant for the authorities in charge of mosquito control operations, especially at the finding locations where high probability of overwintering is predicted. At these sites, eggs would be expected to survive the winter, larvae will be expected to emerge during the mosquito season in the next year, and as a consequence mosquito control will be advised to prevent establishment of the species. If an introduction of *Ae. albopictus* is found, special attention should also be taken in urban and peri-urban areas, where the species may inhabit artificial containers and catch basins as breeding sites. The variability of microclimate among sites in complex urban conditions could provide more favourable conditions for the species, and thus those areas should be placed under intensive surveillance. Experiences from the invasion in Europe tell us that once this species has colonized an area, eradication might be difficult or impossible to achieve [62, 65].

## Conclusions

This study provides suitability maps for *Ae. albopictus* with particular reference to the Netherlands using the state-of-the-art in models of occurrence with environmental data. Results obtained using MaxEnt and LST models for predicting the suitability for establishment of *Ae. albopictus* are different. MaxEnt is the most restrictive model for the species, with winter temperatures reducing the probability of establishment. Based on the LST model, the current climatic conditions hamper the successful overwintering of eggs of *Ae. albopictus* and their survival as adults in many areas of the country, but in warm years with mild winters, several parts of the Netherlands offer climatic conditions suitable for developing populations. Our results for the year 2014 suggest that such situations do occur, including at locations of used tire companies where we found the species outdoors. For this reason, risk-based surveillance is crucial for promptly detecting *Ae. albopictus* at risk locations and engage mosquito control actions.

## Supplementary information

**Additional file 1: Figure S1.** Presence records and species distribution map obtained with *Aedes albopictus* Maxent model. **Figure S2.** Relationship between nineteen European bioclimatic variables (http://www.worldclim.com) for occurrence records buffers of 200 km of radius with histogram and Kernel density (diagonal), Pearson’s correlation coefficient (*r*) and its significance (above the diagonal) and scatterplot and linear regression (below the diagonal). *P ≤ 0.05, **P ≤ 0.01, ***P ≤ 0.001. **Figure S3.** Cluster with nineteen European bioclimatic variables (http://www.worldclim.com) for occurrence records buffers of 200 km of radius. **Figure S4.** Map with eleven presence records of *Aedes albopictus* in the Netherlands. The sites are Almere, Assen, Emmeloord, Etten-Leur, Hardenberg, Lelystad, Moerdijk, Montfoort, Oosterhout, Oss, Weert. Map by authors. **Figure S5.** Maps showing maximum, median, minimum and standard deviation of habitat suitability obtained with *Aedes albopictus* Maxent model. **Figure S6.** The relative contribution of five environment variables (BIO11, BIO8, BIO7, BIO2, BIO12) to the model according to the regularized training gain of jackknife test in the *Aedes albopictus* Maxent model. Blue bars show the influence of each variable alone and green bars show the performance of the model when the variable is removed from the model. **Figure S7.** Response curves showing the relationships between the probability of presence of *Aedes albopictus* and five environment variables (BIO11, BIO8, BIO7, BIO2, BIO12). **Figure S8.** A scatter plot and the corresponding regression line and regression equation for the relationship between the dependent variable air temperature TG (°C × 0.1) and the independent variable LST (°C × 0.01). *Abbreviations*: *r*, Pearson’s correlation coefficient; R-square linear, coefficient of determination; *P*, *P*-value.

## Data Availability

Data supporting the conclusions of this article are included within the article and its additional file. The datasets used and analyzed during the present study are available from the corresponding author upon reasonable request.

## Abbreviations

ECDC: European Centre for Disease Prevention and Control
GDD: accumulated daily growing degree days
IMS: invasive mosquito species
KNMI: Royal Netherlands Meteorological Institute
LST: land surface temperature
MaxEnt: maximum entropy approach
MODIS: moderate resolution imaging spectroradiometer
