# Supplementary material for: Habitat suitability modelling to assess the introductions of Aedes albopictus (Diptera: Culicidae) in the Netherlands
Source: Parasit Vectors. 2020 Apr 26;13:217. doi: 10.1186/s13071-020-04077-3 (PMC7184689; doi:10.1186/s13071-020-04077-3)
Supplement: Supplementary file 1 — Additional file 1: Figure S1. Presence records and species distribution map obtained with Aedes albopictus Maxent model. Figure S2. Relationship between nineteen European bioclimatic variables (http://www.worldclim.com) for occurrence records buffers of 200 km of radius with histogram and Kernel density (diagonal), Pearson’s correlation coefficient (r) and its significance (above the diagonal) and scatterplot and linear regression (below the diagonal). *P ≤ 0.05, **P ≤ 0.01, ***P ≤ 0.001. Figure S3. Cluster with nineteen European bioclimatic variables (http://www.worldclim.com) for occurrence records buffers of 200 km of radius. Figure S4. Map with eleven presence records of Aedes albopictus in the Netherlands. The sites are Almere, Assen, Emmeloord, Etten-Leur, Hardenberg, Lelystad, Moerdijk, Montfoort, Oosterhout, Oss, Weert. Map by authors. Figure S5. Maps showing maximum, median, minimum and standard deviation of habitat suitability obtained with Aedes albopictus Maxent model. Figure S6. The relative contribution of five environment variables (BIO11, BIO8, BIO7, BIO2, BIO12) to the model according to the regularized training gain of jackknife test in the Aedes albopictus Maxent model. Blue bars show the influence of each variable alone and green bars show the performance of the model when the variable is removed from the model. Figure S7. Response curves showing the relationships between the probability of presence of Aedes albopictus and five environment variables (BIO11, BIO8, BIO7, BIO2, BIO12). Figure S8. A scatter plot and the corresponding regression line and regression equation for the relationship between the dependent variable air temperature TG (°C × 0.1) and the independent variable LST (°C × 0.01). Abbreviations: r, Pearson’s correlation coefficient; R-square linear, coefficient of determination; P, P-value. [file 13071_2020_4077_MOESM1_ESM.docx]

**Additional file 1**


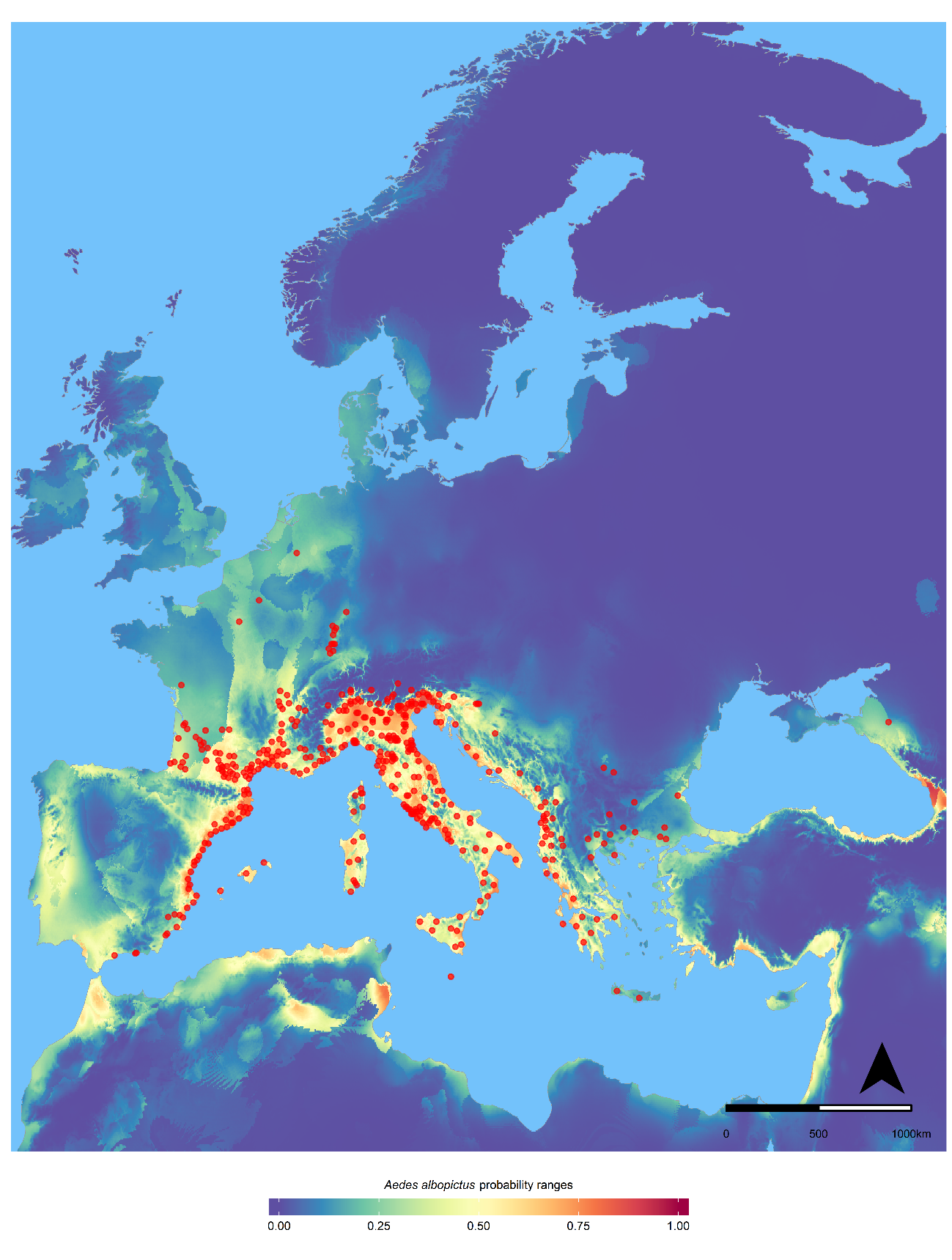


**Figure S1.** Presence records and species distribution map obtained with *Aedes albopictus* Maxent model.


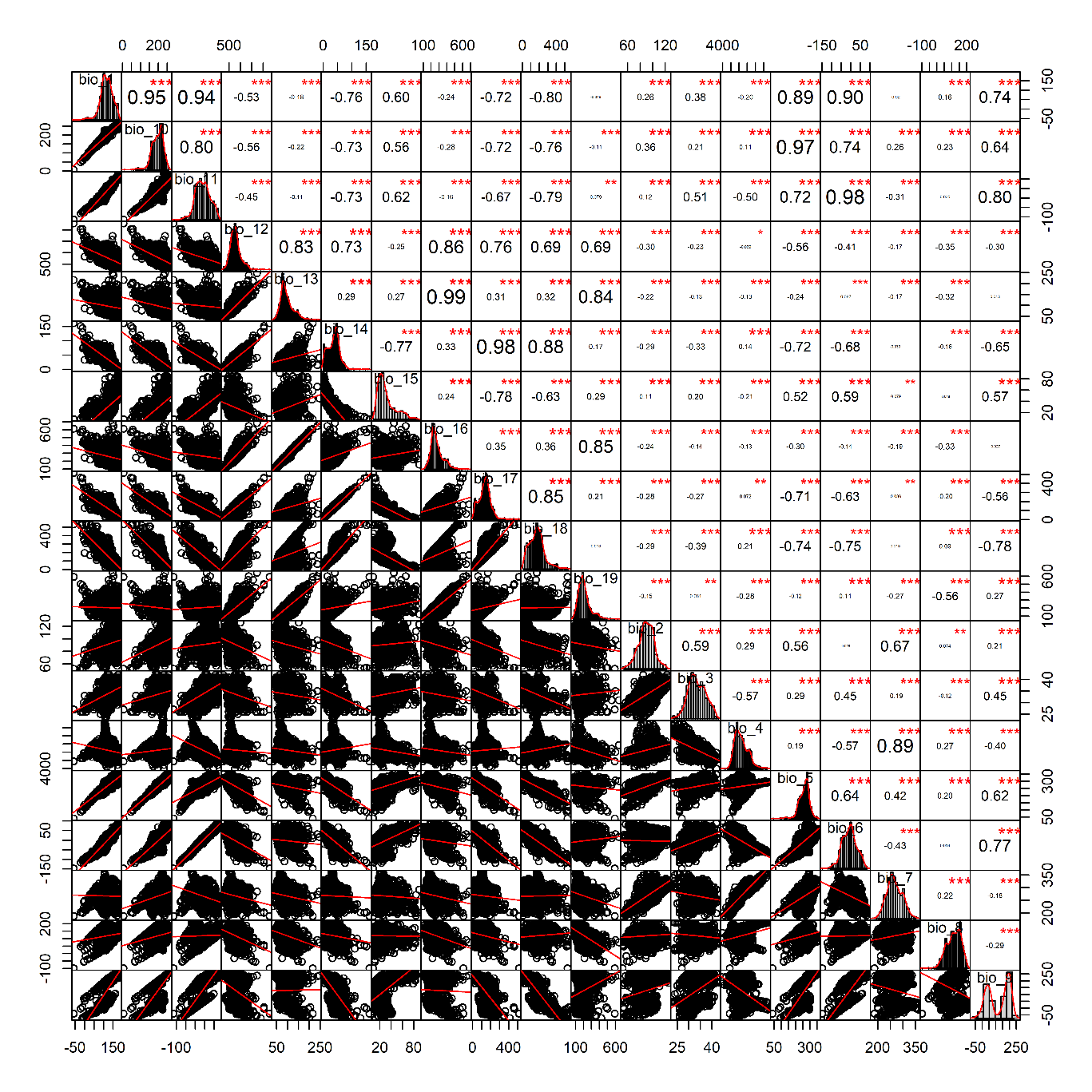


**Figure S2.** Relationship between nineteen European bioclimatic variables ([www.worldclim.com](http://www.worldclim.com)) for occurrence records buffers of 200 km of radius with histogram and Kernel density (diagonal), Pearson correlation coefficient (*r*) and its significance (above the diagonal) and scatterplot and linear regression (below the diagonal). *, *P* ≤ 0.05; **, *P* ≤ 0.01; ***, *P* ≤ 0.001.


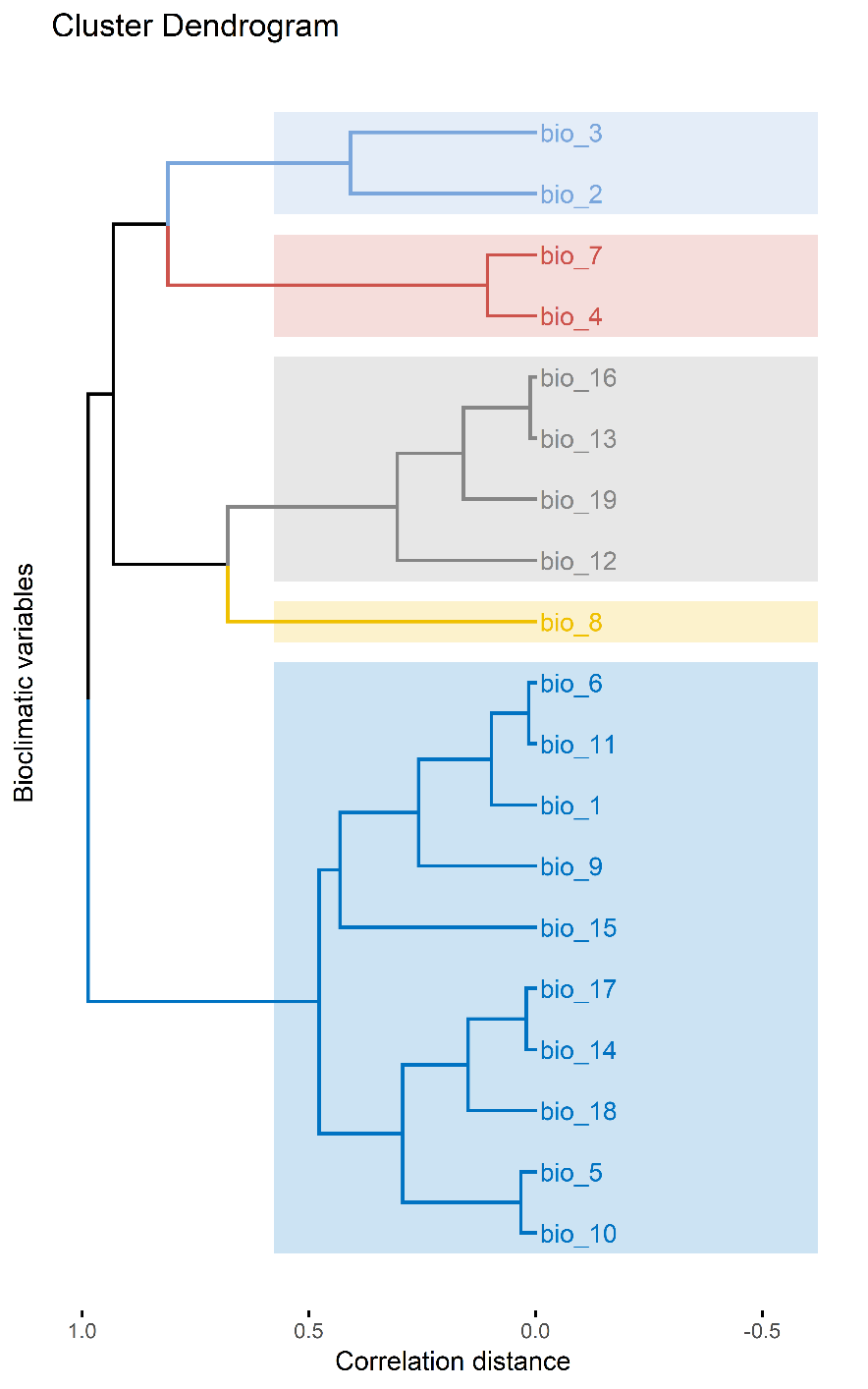


**Figure S3.** Cluster with nineteen European bioclimatic variables ([www.worldclim.com](http://www.worldclim.com)) for occurrence records buffers of 200 km of radius.


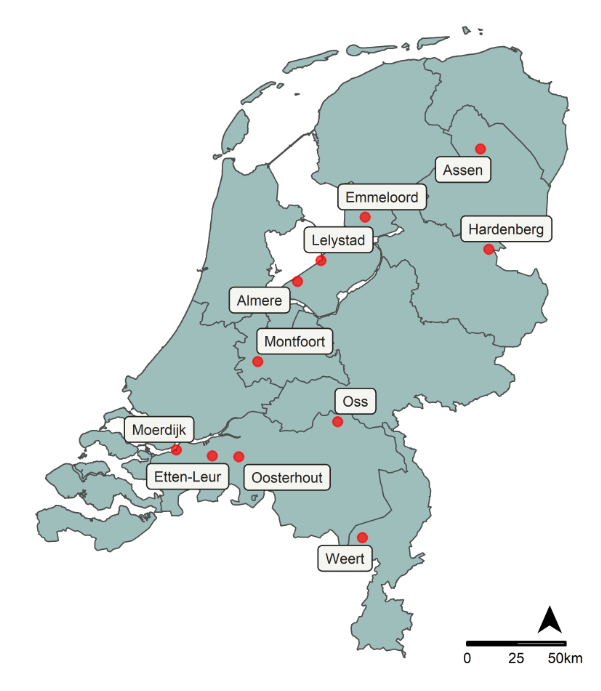


**Figure S4.** Map with eleven presence records of *Aedes albopictus* in Netherlands. The sites are Almere, Assen, Emmeloord, Etten-Leur, Hardenberg, Lelystad, Moerdijk, Montfoort, Oosterhout, Oss, Weert. Map by authors.


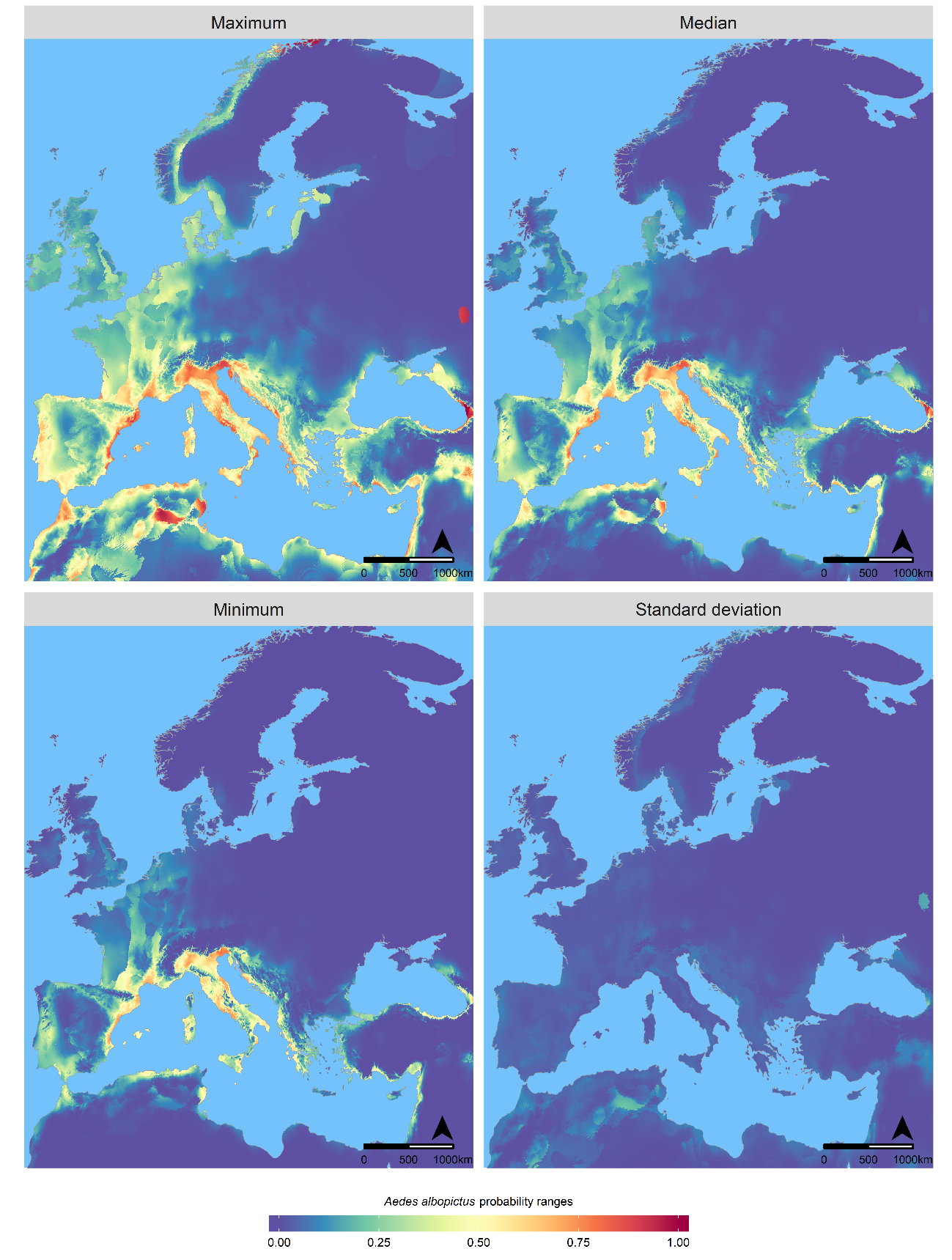


**Figure S5.** Maps showing maximum, median, minimum and standard deviation of habitat suitability obtained with *Aedes albopictus* Maxent model.


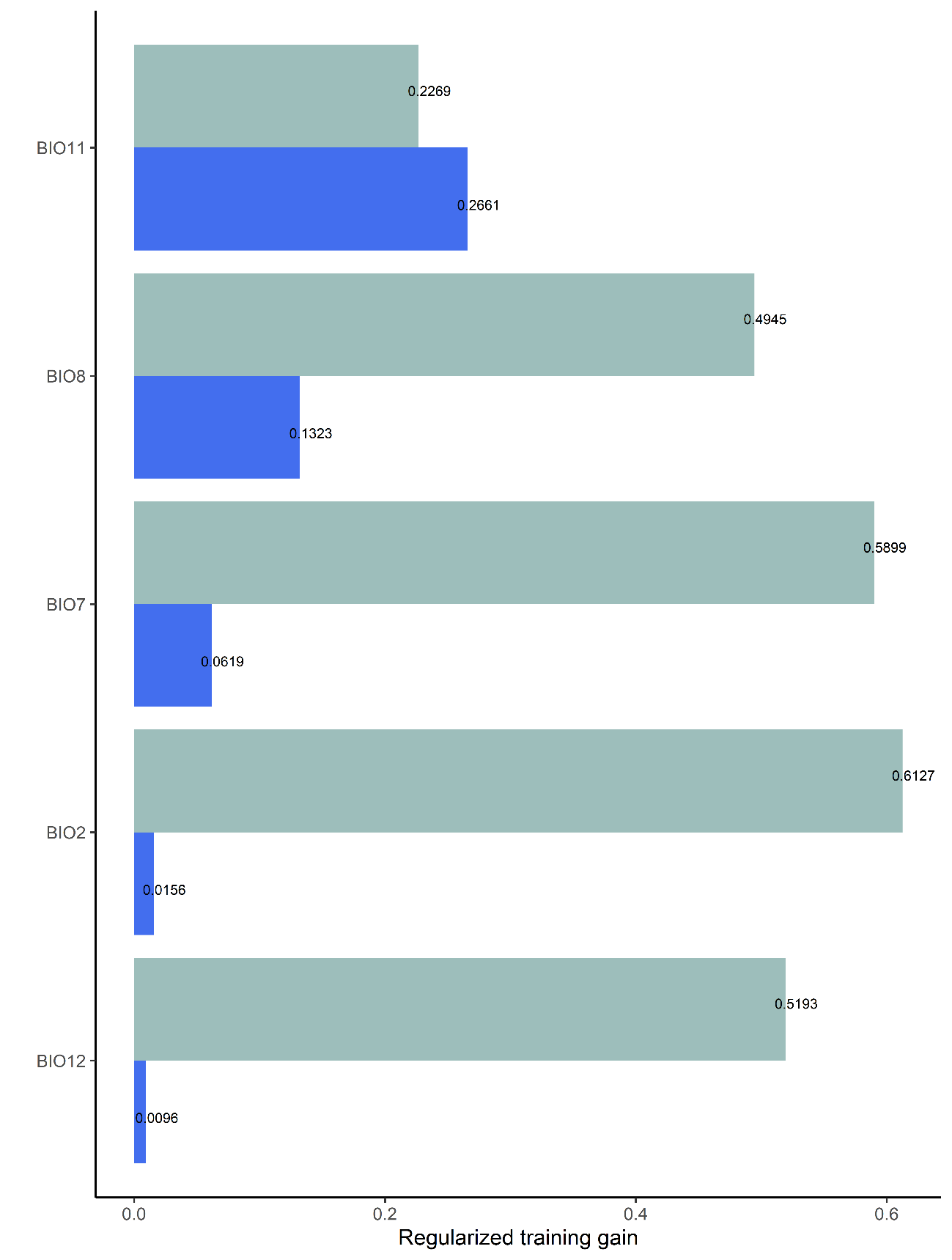


**Figure S6.** The relative contribution of five environment variables (BIO11, BIO8, BIO7, BIO2, BIO12) to the model according to the regularized training gain of jackknife test in the *Aedes albopictus* Maxent model. Blue bars show the influence of each variable alone and green bars show the performance of the model when the variable is removed from the model.


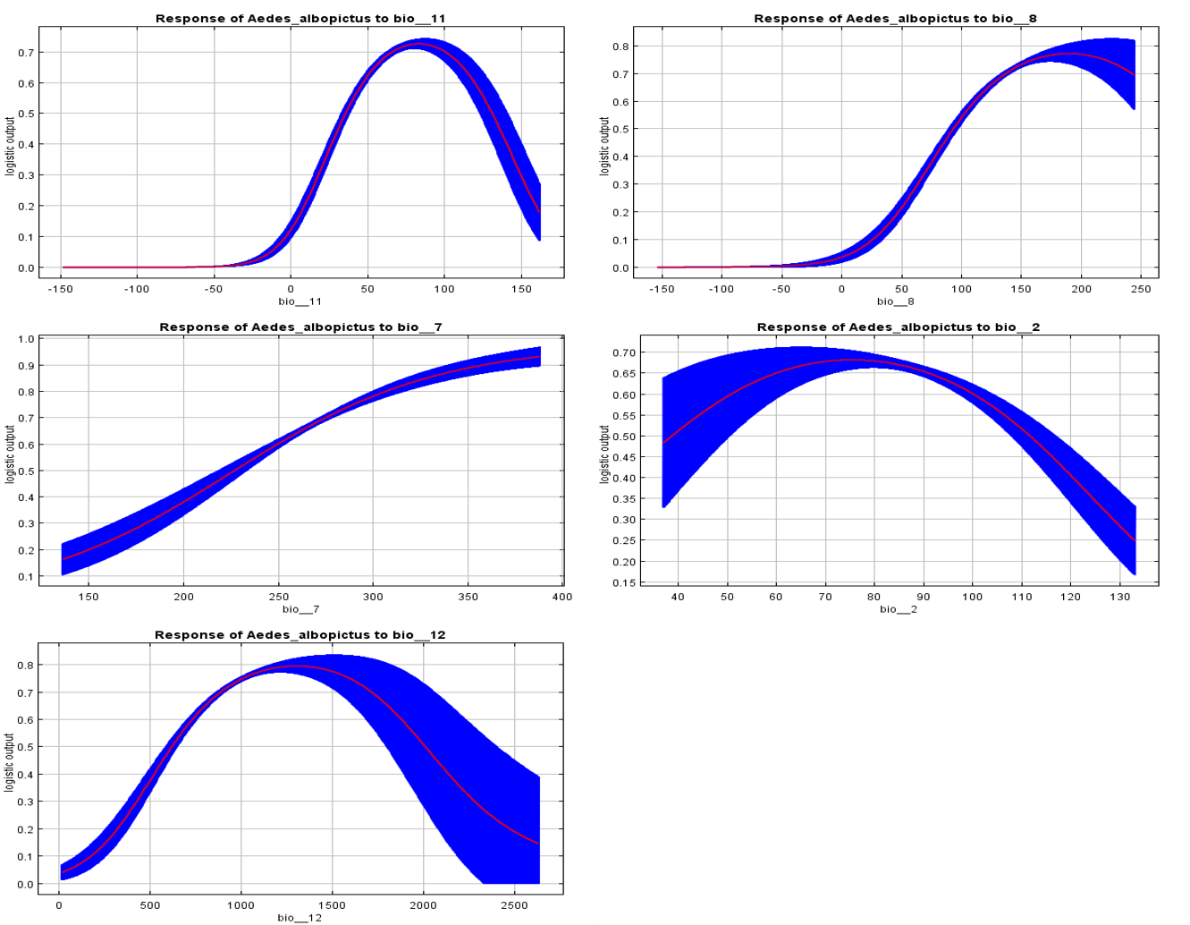


**Figure S7.** Response curves showing the relationships between the probability of presence of *Aedes albopictus* and five environment variables (BIO11, BIO8, BIO7, BIO2, BIO12).


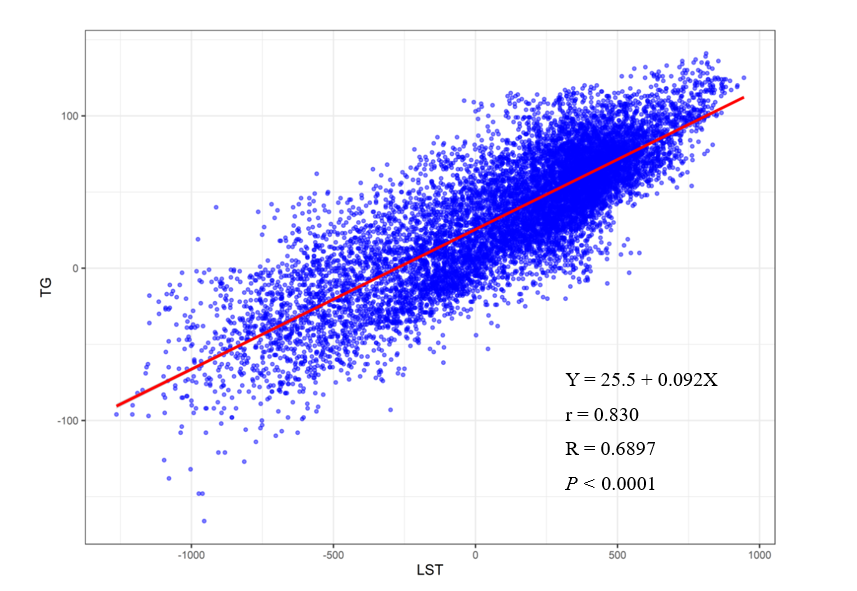


**Figure S8.** A scatter plot and the corresponding regression line and regression equation for the relationship between the dependent variable air temperature TG (ºC*0.1) and the independent variable LST (ºC*0.01). r = Pearsons’s correlation coefficient. R = R-squared linear = coefficient of determination. *P* = P-value.
